# The Persistence of Typhoid Bacilli in Organs for Years

**Published:** 1907-12-07

**Authors:** 


### Illustrative Cases.

### LATENT TYPHOID BACILLI.

It is a remarkable fact that typhoid bacilli, when they cause a suppurative lesion in some organ of the body other than the bowel, are very difficult indeed to eradicate; and it is still more remarkable that, in cases of typhoidal suppuration, the typhoid bacilli seem to have the power to prevent other micro-organisms from growing in the same place. It happens, therefore, that typhoid bacilli are present in pure culture even for years in such suppurative lesions as sometimes follow typhoid fever.

### Empyema of Gall Bladder.

One of the less rare examples of this is in connection with empyema of the gall bladder. Fortunately it is not common for any suppurative lesion to follow typhoid fever; but of all those that are possible, empyema of the gall bladder is as frequent as any. The gall bladder has to be opened and drained. Cultivations from its contents at the time give a pure culture of typhoid bacilli. The sinus goes on discharging for months, tending to close at times, opening up again at others, and sometimes continuing open for years. Cultivations from the bile coming through the sinus give pure cultures of typhoid bacilli all the time. Apparently staphylococci and streptococci find it very difficult to invade a place where the typhoid bacillus is already in possession.

The importance of this in regard to the long-continued danger of the spread of typhoid bacilli to those who are tending the patient is obvious and needs no comment.

### Sub-mammary Abscess.

Empyema of the gall bladder has been given because it is so well known. Examples of a similar state of affairs elsewhere in the body are quite recognised, however, and their possibility should be borne in mind. For instance, a woman recently came up complaining of severe pain in her breast. Examination showed that this was due to an abscess, and at the operation this abscess was found to be sub-mammary. The cause of the suppuration was not clear at first, but cultivations were taken from the pus as usual, and the report came back from the laboratory that the cultures gave a pure growth of typhoid bacilli; this was confirmed by testing the bacteria with the serum of a patient suffering from typhoid fever. Further examination of the history of the patient herself showed that she had had an illness which was not diagnosed, but which was very probably typhoid fever, six months before she came up complaining of her breast. The sub-mammary abscess was due to a post-typhoidal perichondritis or periostitis in connection with a rib. The sinus was a very long time in healing, and throughout this time the pus that came from it gave pure cultures of bacillus typhosus. Special precautions had to be taken, therefore, in dealing with the soiled dressings and with the patient's clothes; indeed, with everything that could possibly be contaminated with the typhoid bacilli coming from the abscess.

### Importance of Cultivating all Pus.

The case shows the great importance of having cultivations made from the pus in any abscess; the determination of the organism at work is not merely of scientific interest, as is apt to be thought; there are many cases in which the bacteriological findings may be of immense practical importance.

### Other Lesions.

We do not propose to enumerate all the possible suppurative lesions in which typhoid bacilli may persist for months or years in pure culture. We may simply mention three other rather important ones—namely, subcutaneous boils, otorrhœa, and pleural empyema—in any of which bacillus typhosus may be present and yet may be overlooked. The conditions are not very common after typhoid fever, it is true, but, unfortunately, that is just why the fact of their being possibly typhoidal is apt to be forgotten. When they occur immediately after the typhoid fever, the organism at work will be in one's mind; but when they are delayed, as in the case of the sub-mammary abscess mentioned above, the connection is not at once obvious. Indeed, in the case mentioned the typhoid fever was not diagnosed when it was in progress; it was only realised as having been typhoid fever when the nature of the organism in the abscess was determined months afterwards.

### Pyonephrosis due to Typhoid Bacilli.

Typhoidal suppuration in the kidney is distinctly rare, and we give the following case not because the lesion itself is common, but because the patient is a very good example of the long persistence of typhoid bacilli in pure culture for years after the enteric fever. A number of these cases have been recorded by Dr. Hugh H. Young and Dr. Louis C. Lehr in the Johns Hopkins Hospital Reports. The patient was a male who complained of bladder trouble. He had had typhoid fever in August 1893, with one relapse. The fever ran its usual course, and it was of ordinary severity. The urine at the time was not noticed to be abnormal, and the patient was discharged in apparently good health. He was not seen again until July 1897, when he returned complaining of a urethral discharge of two weeks' duration, with dysuria and increased frequency of micturition, apparently gonorrhœal. The urethritis cleared up in a few weeks, but for the next three years, though no other symptoms were present, he frequently saw "corruption" in his urine, which was always milky in appearance. He continued his usual work. He stated that although the gonorrhœal urethritis was the cause of his seeking advice in 1897, his urine had been milky ever since his convalescence from typhoid fever in 1893.

In 1898 the urine was very cloudy, with a dirty grey deposit of pus on standing. Bacteriological examination of a catheter specimen was made with extreme care; the result was controlled by the serum test, and it became clear that the urine abounded with typhoid bacilli in pure culture, no other organisms being present. The condition was now diagnosed as chronic cystitis due to bacillus typhosus; the kidneys did not appear to be diseased. There was little pain, and no hæmaturia. It is worthy of note that the patient's blood serum still gave a positive Widal's reaction in dilutions up to 1 in 500. This is interesting because, as Dr. Herbert French has shown in the "Guy's Hospital Reports," 1907, the Widal's test rapidly becomes negative again after typhoid fever when there have been no complications. The patient now began to be more ill, suffering from malaise, nausea, chronic constipation, and pain in the loins, though he was not confined to bed. He was treated with urotropine, vesical lavage, and so forth, and improved for a time, but always relapsed; until, in September 1903, he had lost twenty-three pounds' weight in three weeks, had severe pains in the loins, much pyuria, and was too weak to work. He became uræmic, and died ten years later.

Autopsy.

The only organs that were materially affected were the kidneys, ureters, and bladder. There were no ulcers in the intestine. The kidneys were very large, and contained numerous chronic abscesses full of thick creamy pus; there were concretions in the pyogenic membranes in many places. There was great diminution in the renal tissue proper; the pelves were dilated and inflamed, the ureters were much dilated, and the bladder was contracted, thick-walled, and in a state of chronic cystitis. Cultivations from the renal pus gave pure cultures of typhoid bacilli, confirmed by Widal's serum test. The case, therefore, is an excellent example of the two points we wish to make:—

1. That typhoid bacilli may persist for many years in foci of post-typhoidal suppuration.

2. That in such cases secondary infection with cocci is not usual.

